# An Assay to Determine Mechanisms of Rapid Autoantibody-Induced Neurotransmitter Receptor Endocytosis and Vesicular Trafficking in Autoimmune Encephalitis

**DOI:** 10.3389/fneur.2019.00178

**Published:** 2019-03-01

**Authors:** Elsie Amedonu, Christoph Brenker, Sumanta Barman, Julian A. Schreiber, Sebastian Becker, Stefan Peischard, Nathalie Strutz-Seebohm, Christine Strippel, Andre Dik, Hans-Peter Hartung, Thomas Budde, Heinz Wiendl, Timo Strünker, Bernhard Wünsch, Norbert Goebels, Sven G. Meuth, Guiscard Seebohm, Nico Melzer

**Affiliations:** ^1^Myocellular Electrophysiology and Molecular Biology, Institute for Genetics of Heart Diseases, University of Muenster, Muenster, Germany; ^2^Department of Neurology, University of Muenster, Muenster, Germany; ^3^Centre of Reproductive Medicine and Andrology, University of Muenster, Muenster, Germany; ^4^Department of Neurology, Universitätsklinikum and Center for Neurology and Neuropsychiatry LVR Klinikum, Heinrich Heine University Duesseldorf, Duesseldorf, Germany; ^5^Institute for Physiology I, University of Muenster, Muenster, Germany; ^6^Institute for Pharmaceutical and Medical Chemistry, University of Muenster, Muenster, Germany

**Keywords:** autoimmune encephalitis, N-Methyl-D-aspartate receptors, cross-linking, endocytosis, vesicular trafficking, exocytosis, autoantibodies

## Abstract

N-Methyl-D-aspartate (NMDA) receptors (NMDARs) are among the most important excitatory neurotransmitter receptors in the human brain. Autoantibodies to the human NMDAR cause the most frequent form of autoimmune encephalitis involving autoantibody-mediated receptor cross-linking and subsequent internalization of the antibody-receptor complex. This has been deemed to represent the predominant antibody effector mechanism depleting the NMDAR from the synaptic and extra-synaptic neuronal cell membrane. To assess in detail the molecular mechanisms of autoantibody-induced NMDAR endocytosis, vesicular trafficking, and exocytosis we transiently co-expressed rat GluN1-1a-EGFP and GluN2B-ECFP alone or together with scaffolding postsynaptic density protein 95 (PSD-95), wild-type (WT), or dominant-negative (DN) mutant Ras-related in brain (RAB) proteins (RAB5WT, RAB5DN, RAB11WT, RAB11DN) in HEK 293T cells. The cells were incubated with a pH-rhodamine-labeled human recombinant monoclonal GluN1 IgG1 autoantibody (GluN1-aAb^pH−rhod^) genetically engineered from clonally expanded intrathecal plasma cells from a patient with anti-NMDAR encephalitis, and the pH-rhodamine fluorescence was tracked over time. We show that due to the acidic luminal pH, internalization of the NMDAR-autoantibody complex into endosomes and lysosomes increases the pH-rhodamine fluorescence. The increase in fluorescence allows for mechanistic assessment of endocytosis, vesicular trafficking in these vesicular compartments, and exocytosis of the NMDAR-autoantibody complex under steady state conditions. Using this method, we demonstrate a role for PSD-95 in stabilization of NMDARs in the cell membrane in the presence of GluN1-aAb^pH−rhod^, while RAB proteins did not exert a significant effect on vertical trafficking of the internalized NMDAR autoantibody complex in this heterologous expression system. This novel assay allows to unravel molecular mechanisms of autoantibody-induced receptor internalization and to study novel small-scale specific molecular-based therapies for autoimmune encephalitis syndromes.

## Introduction

Most of the glutamatergic signaling mechanisms in the central nervous system (CNS) rely on the binding of this neurotransmitter (NT) to specific glutamate receptors (GluRs). Ionotropic ligand-gated ion channels (iGluRs) and metabotropic G protein-coupled receptors (mGluRs) mediate fast and slow glutamatergic excitatory synaptic transmission at synapses between neuronal axons and dendrites (1). The iGluRs include the slow, modulatory N-Methyl-D-aspartate receptors (NMDARs), the fast α-Amino-3-hydroxy-5-methyl-4-isoxazolepropionic acid receptors (AMPARs) (2), and the Kainate receptors (KARs), which typically do not contribute to baseline synaptic transmission.

Functional adult neuronal NMDARs are hetero-tetrameric complexes, formed predominantly by two GluN1 and two GluN2 subunits (3). The subunits share a similar membrane topology, i.e., four transmembrane domains (M1–M4), a reentrant membrane loop between M3 and M4 domains, and long extracellular N- and intracellular C-termini (relatively short for GluN1) (4). Hallmarks of NMDARs include voltage-sensitive block by extracellular Mg^2+^, slow current kinetics, and high Ca^2+^ permeability (1). Thereby, NMDARs serve a crucial function in synaptic plasticity (expressed as a change in receptor number and functional properties), learning, and memory. These processes start with the release of glutamate from presynaptic axon terminals and the subsequent binding together with the co-agonist glycine mainly to postsynaptic NMDARs. Postsynaptic NMDARs, in turn, are associated with and regulated by several proteins that together constitute the postsynaptic density (PSD), an elaborate complex of interlinked proteins and elements of the cytoskeleton.

Neuronal glutamate receptor trafficking is a multi-step process that involves protein synthesis at the dendritic tree of the postsynaptic neuron, receptor subunit quality control and assemblage in the endoplasmic reticulum (ER), processing in the Golgi apparatus (GA), vesicular packaging in the Golgi complex (GC), subsequent *vertical trafficking* to the neuronal cell surface membrane and anchorage at the PSD, *lateral trafficking* into and out of the PSD, as well as the internalization (endocytosis), subsequent neuronal surface membrane reinsertion (exocytosis) carried out by endosomes (*vertical trafficking*) or degradation carried out by lysosomes (5). At each step of the trafficking process, NMDARs associate with specific partner proteins that allow for their maturation and/or transportation (4).

Glutamate receptors are major targets in autoimmune encephalitis syndromes (6, 7), in which autoantibodies of the immunoglobulin (Ig) G type target iGluRs like NMDARs (8) and AMPARs (9) as well as mGluRs like metabotropic glutamate receptor 1 (mGluR1) (10) and 5 (mGluR5) (11). These autoantibodies disrupt receptor function, cross-link receptors leading to internalization of the antibody-receptor complex (9, 12–15), and activate complement depending on the autoantibody, its IgG subclass, and the complement concentration in the cerebrospinal fluid.

NMDAR autoantibodies are of the IgG 1 or 3 subtypes and can directly affect the gating of the receptor (16). Residues N^368^/G^369^ in the extracellular domain of the GluN1 subunit of NMDARs may form part of the immunodominant binding region for IgG on the receptor molecule. In single-channel recordings, antibody binding to the receptor instantly caused more frequent openings and prolonged open times of the receptor (16). Moreover, NMDAR autoantibodies caused selective and reversible decrease in postsynaptic surface density and synaptic anchoring of NMDAR in both glutamatergic and GABAergic rat hippocampal neurons by disrupting the interaction of NMDAR with Ephrin-B2 receptors (17), followed by selective NMDAR cross-linking and internalization (13, 14). Consistently, NMDAR antibodies selectively decreased NMDAR-mediated miniature excitatory post-synaptic currents (mEPSCs) without affecting AMPAR-mediated mEPSCs in cultured rat hippocampal neurons (13).

In cultured rat hippocampal neurons, once internalized, antibody-bound NMDAR traffic through recycling endosomes and lysosomes, but do not induce compensatory changes in glutamate receptor gene expression (14). The internalized antibody-receptor complexes co-localize rather with RAB11-positive recycling endosomes than with Lamp1-positive lysosomes suggesting subsequent recycling and exocytosis (14). The process of NMDAR internalization plateaus after 12 h, reaching a steady state that persists throughout the duration of the antibody treatment (14), likely reflecting a state of equilibrium between the rate of receptor internalization and the rate of receptor (re-)insertion from different compartments into the surface membrane (14).

Probably due to the lack of blood-brain barrier disruption in NMDAR encephalitis and subsequent lack of relevant complement concentrations in the cerebrospinal fluid, as well as internalization of NMDAR together with the autoantibodies, no complement depositions or major neuronal loss could be detected in biopsy specimens of patients with NMDAR encephalitis, despite large numbers of intracerebral autoantibody-secreting plasma cells (18, 19). Indeed, fully reversible impairment of behavior and memory occurs in mice receiving passive intrathecal transfer of NMDAR autoantibodies (20, 21) that is prevented by co-application of ephrin (22).

The effects on receptor-mediated currents are rather small in heterologous expression systems and do not allow for mechanistic studies on autoantibody-induced neurotransmitter receptor internalization and trafficking in anti-NMDAR encephalitis and other forms of autoimmune encephalitis (21). Thus, the aim of this study was to develop an assay suitable to study in molecular detail the mechanism of autoantibody-induced NMDAR endocytosis, vesicular trafficking, and exocytosis and potentially to study novel small-scale specific molecular-based therapies for autoimmune encephalitis syndromes.

## Materials and Methods

### Construction of NMDAR Expression Vectors

NMDAR constructs were kindly provided by Prof. Michael Hollmann, Receptor Biochemistry, Faculty of Chemistry and Biochemistry, Ruhr University Bochum, Germany. cDNAs encoding the rat GluN1-1a and GluN2B NMDAR subunits were sub-cloned into the pEGFP-N1 and pECFP-N1 mammalian expression vectors, respectively. To allow for the visualization of the subunits, enhanced cyan fluorescent protein (ECFP), and enhanced green fluorescent protein (EGFP) were inserted in-frame at the N-terminus of the subunits. The subunit-containing plasmids were amplified via growth in *E. coli* followed by purification based on a modified alkaline lysis procedure (QIAGEN Miniprep kit).

The generation of PSD-95 as well as WT and DN RAB5 and RAB11 expression vector constructs has been described elsewhere (5, 23).

### HEK 293T Cell Co-transfection

HEK 293T cells were cultured in growth media comprising high-glucose Dulbecco's modified Eagle's medium (DMEM) supplemented with 10% fetal bovine serum (FBS), non-essential amino acids (NEAA), Pen-Strep, and 2 mM glutamine. Two days prior to transfection, exponentially growing cells were seeded on poly-D-lysine-coated glass bottom 96-well-plates to a density of approximately 5.0–8.0 × 10^5^/well. Two hours prior to co-transfection, the culture medium was replaced with fresh culture medium. HEK 293T cells were then transiently co-transfected with the cDNAs (0.25 μg GluN1-1a-EGFP and 0.25 μg GluN2B-ECFP) encoding the NMDAR subunits as well as PSD-95, or WT, or DN RAB proteins using the FuGene HD (Promega Corporations) transfection technique according to manufacturer's instructions or left untransfected. 24 h post co-transfection, cells were seeded onto poly-D-lysine-coated glass bottom 96-well-plates. Confocal laser-scanning microscopy was used to quantify cell-surface NMDAR density (×63 glycerol objective; TCS-SP5 Leica- Microsystems, Germany).

### pH-rhodamine Labeling of a Human Recombinant Monoclonal GluN1 Autoantibody

Generation of a recombinant human monoclonal GluN1 autoantibody (GluN1-aAb) engineered from clonally expanded intrathecal plasma cells of a patient with anti-NMDAR encephalitis has recently been described (21). Labeling of GluN1-aAb was performed using a pHrodo™ Red Microscale Labeling Kit (Thermofisher Scientific) according to the recommendations of the supplier. Briefly, 100 μL of GluN1-aAb solution (1 mg/mL in PBS) were transferred to a “component D” containing reaction tube and supplemented with 10 μL of 1M sodium bicarbonate. pHrodo red succinimidyl ester was dissolved in 10 μL of DMSO. From the resulting solution, 0.70 μL (as calculated according to the equation 1 of the labeling kit's protocol) was added to the reaction tube containing the pH-adjusted GluN1-aAb. This reaction mixture was incubated for 15 min at RT to allow conjugation. For separation from unbound dye, the reaction mix was spun through a resin-containing column (provided with the kit) at 1,000 g for 5 min. The purified pH-rhodo red labeled GluN1-aAb (GluN1-aAb^pH−rhod^) was recovered from the collection tube and stored aliquoted at −20°C, while unbound dye remained in the resin.

### Co-incubation of GluN1-aAb^pH-rhod^ With HEK 293T Cells Expressing GluN1-1a-EGFP/GluN2B-ECFP NMDARs and Fluorescence Intensity Analysis

The rhodamine fluorophore possesses a well-known pH- and temperature-dependent fluorescence quantum yield (24), which decreases linearly as pH and temperature increases (25). These physicochemical properties needed to be considered in our experimental setting.

Untransfected or co-transfected HEK 293T cells from the same 96-well-plate were incubated with GluN1-aAb^pH−rhod^ at a concentration of 4 μg/ml in phosphate-buffered saline (PBS, pH 7.4) for 2 min. After that, unbound GluN1-aAb^pH−rhod^ was washed-out by superfusing 96-wells with PBS to yield cell-bound GluN1-aAb^pH−rhod^ fluorescence. Wells on the same 96-well-plate without cells incubated with GluN1-aAb^pH−rhod^ at a concentration of 4 μg/ml in PBS without wash-off served as control. All incubations were conducted at 4°C on ice to prevent endocytosis prior to recordings.

Subsequently, fluorescence of GluN1-EGFP and GluN1-aAb^pH−rhod^ was excited at 480 and 520 nm and detected at 510 nm (confocal imaging) and 580 nm (plate reader), respectively, verifying GluN1-1a-EGFP expression and presence of the GluN1-aAb-bound rhodamine fluorescence (GluN1-aAb^pH−rhod^).

Subsequently, the temperature was increased rapidly from 4 to 30°C to allow for endocytosis, and pH-rhodamine-fluorescence was repetitively excited at 520 nm and the emission was detected at 580 nm. The overall pH-rhodamine fluorescence decreased exponentially with time reaching a steady state after approximately 500 s mainly reflecting the known temperature-dependent fluorescence quantum yield of rhodamine under all experimental conditions.

The steady state pH-rhodamine-fluorescence intensity at 580 nm after 500 s of HEK 293T cells expressing GluN1-1a-EGFP/GluN2B-ECFP NMDARs was significantly higher compared to untransfected HEK 293T cells and served as a cumulative measure of endocytosis of GluN1-aAb^pH−rhod^ bound to the NMDAR with subsequent acidification within endosomes and/or lysosomes and exocytosis. This allowed for mechanistic studies in HEK 293T cells expressing GluN1-1a-EGFP/GluN2B-ECFP NMDARs co-transfected with scaffolding protein PSD-95 as well as RAB5WT and RAB5DN (mediating vesicle endocytosis) and RAB11WT or RAB11DN (mediating vesicle exocytosis).

### Statistical Analysis

Data was analyzed using Origin 9 (OriginLab Corporation). One-way ANOVA followed by multiple pair-wise comparisons with Bonferroni's *post-hoc* correction was used to statistically analyze differences in fluorescence intensity; *p* ≤ 0.05 were considered as significant; data in figures were expressed as mean ± SEM. All experiments were performed in triplicates.

## Results

To assess in detail the molecular mechanisms of NMDAR autoantibody-induced NMDAR endocytosis, vesicular trafficking, and exocytosis we transiently expressed rat GluN1-1a-EGFP and GluN2B-ECFP alone or together with PSD-95 or with WT- or DN-mutant RAB proteins (RAB5WT, RAB5DN, RAB11WT, RAB11DN) in HEK 293T cells. As a control, HEK 293T cells were left untransfected.

The cells were incubated with a pH-rhodamine-labeled human recombinant monoclonal GluN1 IgG1 autoantibody [GluN1-aAb^pH−rhod^, (21)].

**Figure 1:**
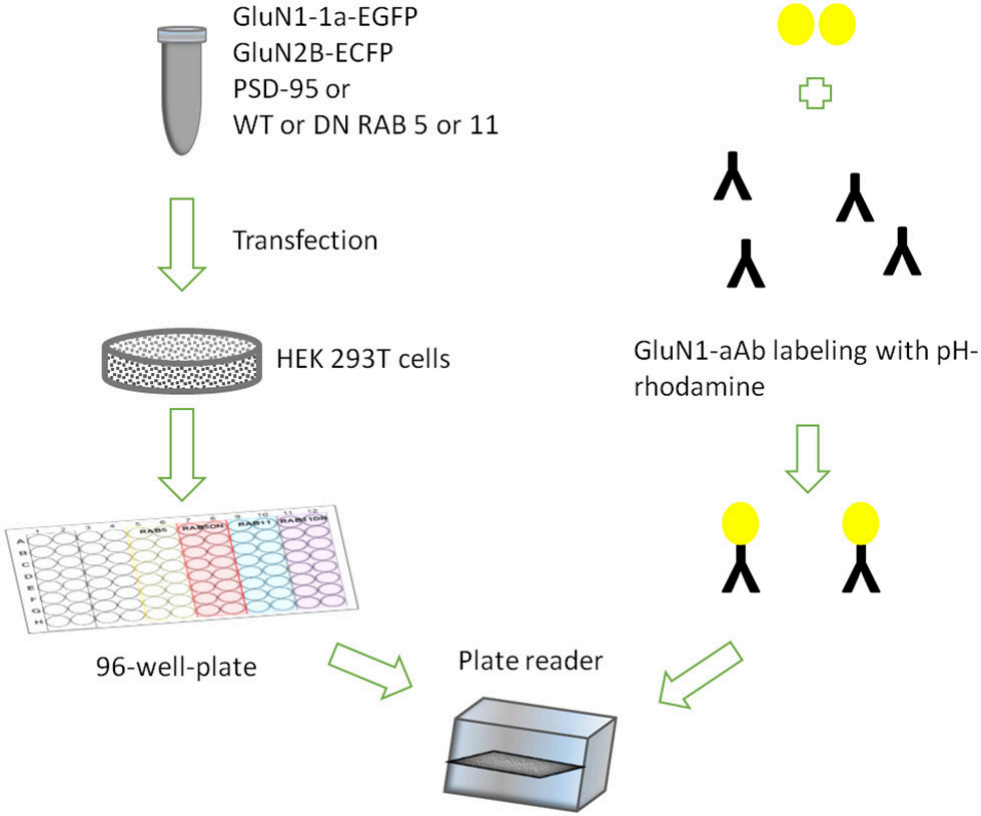
Assay design showing HEK 293T cells transiently expressing rat GluN1-1a-EGFP/GluN2B-ECFP incubated with GluN1-aAb^pH−rhod^ in 96-well-plates under variable conditions in parallel.

We surmised that the pH-rhodamine fluorescence is increased during the ensuing internalization of the NMDAR-autoantibody complex, due to the acidic luminal pH of endosomes and lysosomes. This might allow for mechanistic assessment of endocytosis, vesicular trafficking in both vesicular compartments and exocytosis of the NMDAR-autoantibody complex (for assay design see Figure 1).

**Figure 2:**
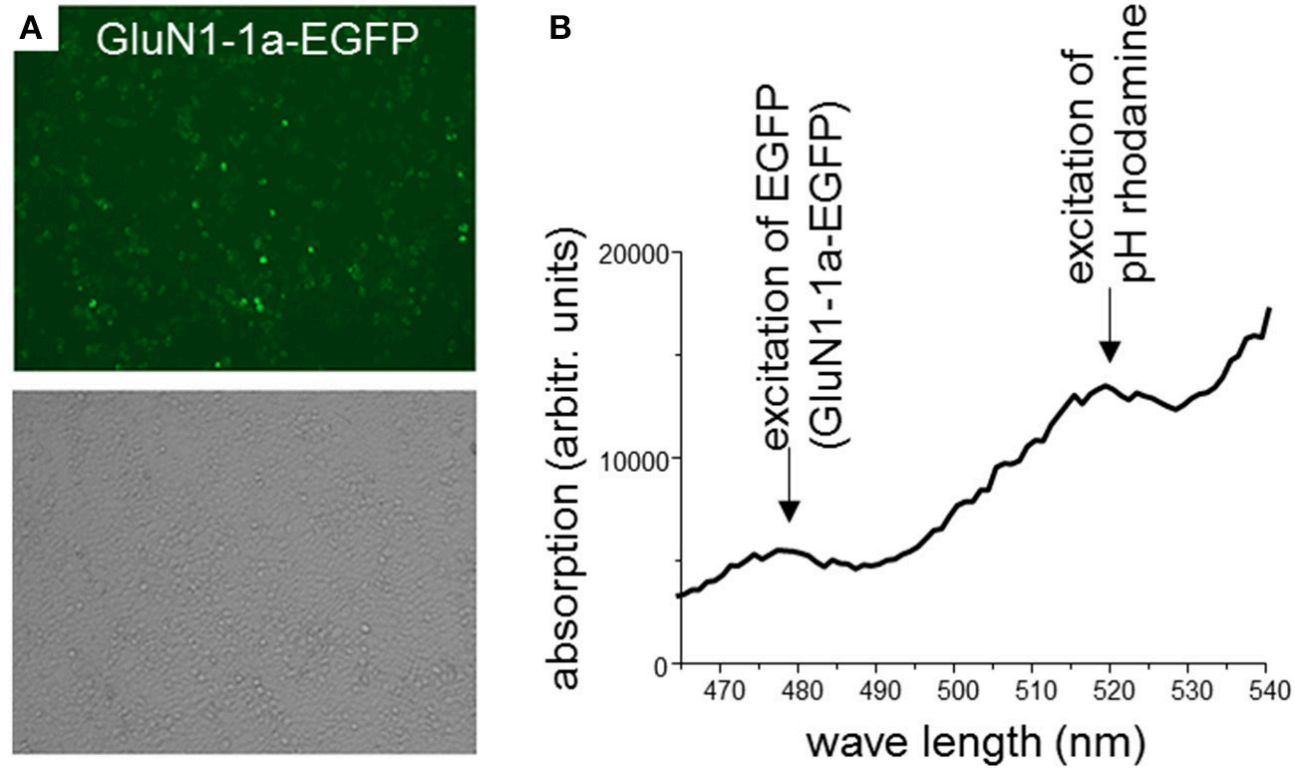
**(A)** HEK 293T cells co-expressing rat GluN1-1a-EGFP and GluN2B-ECFP. 2 days after transfection, expression of fluorescently labeled NMDARs was verified using confocal laser-scanning microscopy. About 70–80% of the cells expressed GluN1-1a-EGFP as subunit putatively targeted by the GluN1-aAb^pH−rhod^ (upper panel) and GluN2B-ECFP (data not shown). Light microscopy demonstrates typical cell densities (lower panel). **(B)** Binding of GluN1-aAb^pH−rhod^ to the NMDAR subunits as determined by the plate reader experiment. HEK 293T cells expressing GluN1-1a-EGFP/GluN2B-ECFP were incubated with GluN1-aAb^pH−rhod^ on ice in 96-well-plates. GluN1-1a-EGFP and GluN1-aAb^pH−rhod^ fluorescence was excited at wavelengths of 480 nm and 520 nm and emission measured at wavelengths of 510 and 580 nm, respectively, verifying the GluN1-1a-EGFP expression and the binding of GluN1-aAb^pH−rhod^.

In a first set of experiments, HEK 293T cells were transiently co-transfected only with rat GluN1-1a-EGFP and GluN2B-ECFP or left untransfected. After 2 days, expression of fluorescently labeled NMDARs was verified using confocal laser-scanning microscopy. About 70–80% of the cells expressed GluN1-1a-EGFP as subunit putatively targeted by the GluN1-aAb^pH−rhod^ (Figure 2A) and GluN2B-ECFP (data not shown). Longer expression times or higher amounts of NMDAR-cDNA for transfections decreased expression levels, supposedly due to cytotoxic effects of pronounced overexpression of NMDARs.

Next, GluN1-1a-EGFP- and GluN2B-ECFP-transfected and untransfected cultured cells were incubated with GluN1-aAb^pH−rhod^. The incubation was performed at 4°C to stop ongoing endocytosis. After that, unbound GluN1-aAb^pH−rhod^ was washed-off. As a control, wells without cells were incubated with GluN1-aAb^pH−rhod^ without wash-off.

Subsequently, GluN1-1a-EGFP and GluN1-aAb^pH−rhod^ fluorescence was excited at 480 nm and 520 nm and measured at 510 and 580 nm, respectively, verifying GluN1-1a-EGFP expression of transfected but not untransfected cells and presence of the GluN1-aAb-bound rhodamine fluorescence (GluN1-aAb^pH−rhod^, Figure 2B).

**Figure 3:**
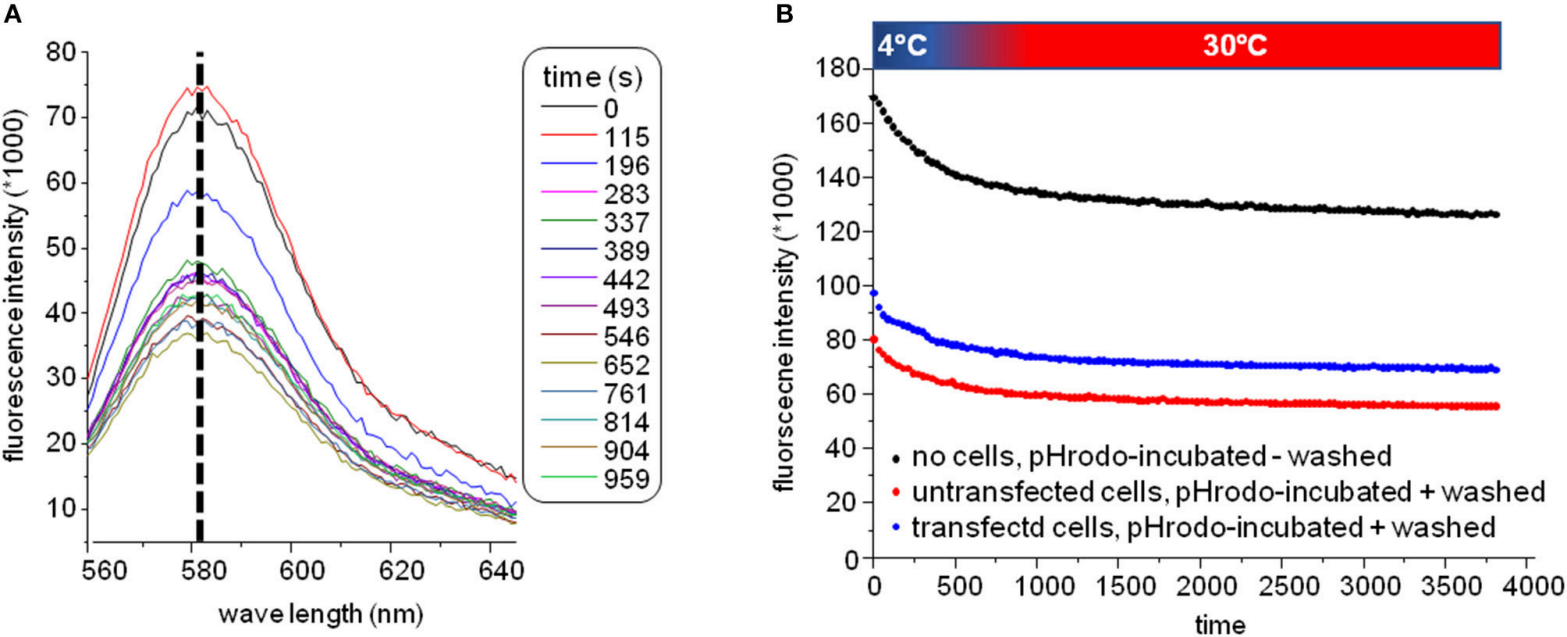
**(A)** Spectra of the GluN1-aAb^pH−rhod^ fluorescence. The overall fluorescence at 580 nm decreased over time reaching a steady state after approximately 500 s mainly reflecting the known temperature-dependent fluorescence quantum yield of rhodamine (24–26) elicited by elevating the temperature for 4–30°C at the beginning of the experiment under all experimental conditions. During this decay, the excitation spectrum did not shift/change indicating that indeed pH-rhodamine-fluorescence was detected throughout the whole experiment. **(B)** Representative time-dependent traces of the GluN1-aAb^pH−rhod^ fluorescence without wash-off of the unbound GluN1-aAb^pH−rhod^ in PBS at pH 7.4 (black trace) and after wash-off of unbound GluN1-aAb^pH−rhod^ in wells seeded with HEK 293T cells expressing GluN1-1a-EGFP/GluN2B-ECFP (blue trace) or untransfected HEK 293T cells (red trace). The steady state pH-rhodamine-fluorescence intensity at 580 nm after 500 s of HEK 293T cells expressing GluN1-1a-EGFP/GluN2B-ECFP NMDARs was significantly higher compared to the background fluorescence of untransfected HEK 293T cells and served as a cumulative measure of endocytosis of GluN1-aAb^pH−rhod^ bound to the NMDAR with subsequent acidification within endosomes and/or lysosomes and exocytosis.

The plate was subsequently transferred to a fluorescence plate reader for time-resolved detection of the pH-rhodamine fluorescence intensity at 30°C. The temperature was increased rapidly from 4–30°C to start endocytosis, and pH-rhodamine-fluorescence was repetitively excited at 520 nm and the emission was detected at 580 nm. The overall rhodamine fluorescence at 580 nm decreased exponentially with time, reaching a steady state after approximately 500 s (Figure 3B) mainly reflecting the known temperature-dependent fluorescence quantum yield of rhodamine (24–26) under all experimental conditions.

Of note, the fluorescence-spectrum did not change over time (Figure 3A), illustrating that pH-rhodamine fluorescence was detected throughout the experiments. Moreover, steady-state fluorescence intensities of empty wells without wash-off of GluN1-aAb^pH−rhod^ were much larger than those of wells with HEK 293T cells illustrating the known background fluorescence of pH rhodamine at neutral pH of 7.4 in PBS (roughly 1/3 of the maximal fluorescence at acidic pH of 4.0) and thus the necessity of the washing step (Figure 3B).

**Figure 4:**
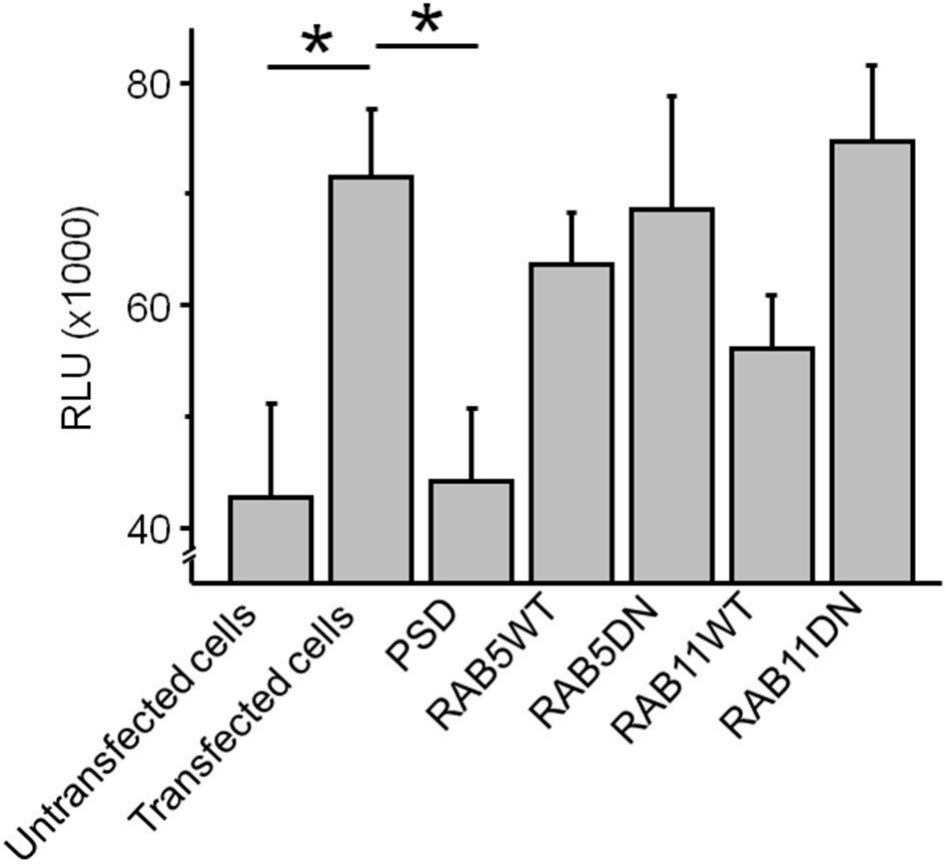
The co-expression of GluN1-1a-EGFP/GluN2B-ECFP NMDARs with the scaffolding protein PSD-95 significantly reduced the cumulative steady state pH-rhodamine-fluorescence intensity at 580 nm after 500 s toward background levels of untransfected HEK 293T cells. In contrast, co-transfection with RAB5WT or RAB5DN (mediating/inhibiting endocytosis) or RAB11 WT or RABDN (mediating/inhibiting exocytosis) did not significantly affect the cumulative steady state pH-rhodamine-fluorescence intensity at 580 nm after 500 s in this heterologous expression system. One-way ANOVA followed by multiple pair-wise comparisons with Bonferroni's *post-hoc* correction was used to statistically analyze differences in fluorescence intensity; *p* ≤ 0.05 were considered as significant (*); data are expressed as mean ± SEM. All experiments were performed in triplicates.

The steady state pH-rhodamine-fluorescence intensity at 580 nm after 500 s of HEK 293T cells expressing GluN1-1a-EGFP/GluN2B-ECFP NMDARs was significantly higher compared to the background fluorescence of untransfected HEK 293T cells (Figure 4) and served as a cumulative measure of endocytosis of GluN1-aAb^pH−rhod^ bound to the NMDAR with subsequent acidification within endosomes and/or lysosomes and exocytosis. This allowed for mechanistic studies in HEK 293T cells expressing GluN1-1a-EGFP/GluN2B-ECFP NMDARs co-transfected with scaffolding protein PSD-95 as well as WT and DN RAB5 (mediating vesicle endocytosis) and RAB11 (mediating vesicle exocytosis).

The co-expression of GluN1-1a-EGFP/GluN2B-ECFP NMDARs with the scaffolding protein PSD-95 significantly reduced the cumulative steady state pH-rhodamine-fluorescence intensity at 580 nm after 500 s toward background levels of untransfected HEK 293T cells (Figure 4). In contrast, co-transfection with RAB5WT or RAB5DN (mediating/inhibiting endocytosis) or RAB11 WT or RABDN (mediating/inhibiting exocytosis) did not significantly affect the cumulative steady state pH-rhodamine-fluorescence intensity at 580 nm after 500 s in this heterologous expression system (Figure 4).

## Discussion

NMDARs are among the most important excitatory receptors in the human brain. NMDAR autoantibodies cause encephalitis by binding to NMDARs, transducing conformational changes and subsequent endocytosis (21, 27). Recently, we showed that pre-incubation for an hour of a recombinant human monoclonal GluN1 autoantibody engineered from clonally expanded intrathecal plasma cells of a patient with anti-NMDAR encephalitis reduced NMDAR-mediated currents recorded from *Xenopus laevis* oocytes by about 20% (21). This result is similar to previous results in *Xenopus laevis* oocytes, showing a time-dependent inhibition of steady-state NMDAR-mediated currents of about 30% within 16 min upon exposure to dialysed sera of patients with anti-NMDAR encephalitis (28). To record NMDAR-mediated currents *in Xenopus laevis* oocytes (and other heterologous expression systems), it is required to use Ca^2+^-free media to block current inactivation (29). This might explain the rather small antibody-mediated action in oocytes (and probably other heterologous expression systems) compared to the pronounced effects on NMDAR expression on neuronal cell surface *in vitro, ex vivo*, and on memory impairment *in vivo* in mice. The Ca^2+^-free recording conditions may cause conformational changes of the NMDAR induced by binding of the antibody or modulate antibody binding itself and thus diminish subsequent receptor cross-linking and internalization.

These effects on receptor-mediated currents in *Xenopus laevis* oocytes (and other heterologous expression systems) do not allow for further mechanistic studies on autoantibody-induced neurotransmitter receptor internalization and trafficking in anti-NMDAR encephalitis and other forms of autoimmune encephalitis (30). Thus, the aim of this study was to develop an assay suitable for this kind of study.

We used a pH-rhodamine labeled single recombinant human GluN1 IgG1 autoantibody [GluN1-aAb^pH−rhod^, (21)]. This monoclonal autoantibody has previously been shown to evoke all effects of natural NMDAR autoantibodies contained in cerebrospinal fluid of patients with anti-NMDAR encephalitis *in vitro* and *in vivo* (21).

We tested the effects of GluN1-aAb^pH−rhod^ incubation on NMDAR endocytosis, trafficking, and exocytosis mechanisms in HEK 293T cells co-transfected with EGFP-tagged GluN1-1a and ECFP-tagged GluN2B subunits alone or together with PSD-95 or WT- or DN-mutant RAB 5 (mediating endocytosis) and 11 (mediating exocytosis) proteins.

Endocytosis, intracellular trafficking, and exocytosis of the antibody-receptor complex is mediated by transporting vesicles with acidic luminal pH. Thus, we took advantage of this fact, as we found that the use of the steady state GluN1-aAb^pH−rhod^ fluorescence in HEK 293T cells expressing GluN1-1a-EGFP/GluN2B-ECFP NMDARs was significantly higher compared to the background fluorescence of untransfected HEK 293T cells. Thus, this steady state fluorescence served as a cumulative measure of endocytosis of GluN1-aAb^pH−rhod^ bound to the NMDAR with subsequent acidification within endosomes and/or lysosomes and exocytosis.

Using this approach we could demonstrate a role for PSD-95 for stabilization of NMDAR in the cell membrane in the presence of NMDAR autoantibodies. This suggests that autoantibody-induced depletion from the cell membrane predominantly affects extra-synaptic NMDARs not associated with PSD-95 and to a lesser extent synaptic NMDARs. This finding is consistent with the notion that autoantibodies through dissociation from clustering ephrinB2 receptors lead to lateral diffusion of synaptic NMDARs within the neuronal cell membrane out to the synapse where they become cross-linked and internalized as extra-synaptic NMDARs (17, 31). Cell membrane stabilization of synaptic NMDARs [displaying pro-survival functions (32)] and internalization of extra-synaptic NMDARs [displaying cell-death promoting functions (32)] is further consistent with the lack of overt neurodegeneration in NMDAR encephalitis despite excitotoxic excessive extracellular levels of glutamate (33–35).

Endocytosis, intracellular trafficking and exocytosis are under the guidance of small G-proteins of the RAB type. The use of functional WT and DN mutants has been previously successful in identification of intracellular trafficking pathways of glutamate receptors (36). We found that co-transfection with WT or DN RAB5 (mediating/inhibiting endocytosis) did not affect the cumulative steady state GluN1-aAb^pH−rhod^ fluorescence intensity, whereas the cumulative steady state GluN1-aAb^pH−rhod^ fluorescence intensity was tentatively lowered by co-transfection with WT RAB11 (mediating exocytosis) and tentatively augmented by co-transfection with RAB11DN (inhibiting exocytosis) compared to expression of NMDARs alone. This lack of overt effects of RAB proteins on endocytosis, trafficking and exocytosis of the antibody-receptor complex in our assay is probably due to the overlay by the temperature-dependent fluorescence decrease the amplitude of which is roughly as large as the steady state amplitude of the pH-dependent fluorescence increase upon internalization of the antibody-receptor complex. These opposing effects hinder detailed kinetic analysis of the autoantibody-induced vertical trafficking of the NMDAR performed here.

Hence, given the necessity of washing-off unbound GluN1-aAb^pH−rhod^ and halting trafficking during that time by lowering the temperature due to the residual rhodamie fluorescence at pH 7.4 in PBS, the use of fluorophores (that inevitably are also concordantly temperature-sensitive) with an optimized pH-dependence i.e., no fluorescence at physiological pH of 7.4 might be better suited for our assay. They would enable synchronic adding of the labeled antibody to the cells cultured in PBS at pH 7.4 at constant temperature of 30°C and thus time-resolved tracking of fluorescence increase upon antibody-receptor internalization.

Taken together, we demonstrate a role for PSD-95 for stabilization of NMDAR in the cell membrane of HEK 293T cells in the presence of NMDAR autoantibodies, while RAB proteins did not exert a significant effect on vertical trafficking of the internalized NMDAR autoantibody complex in this heterologous expression system.

Our assay should be sensitive enough to study novel small-scale specific molecular-based therapies for autoimmune encephalitis that may become feasible as follows:

1. Autoantibody binding to the GluN1 subunit and subsequent induction of conformational changes in the NMDAR could be blocked by antibody fragments, for example. However, with existence of a multitude of autoantibody epitopes within the NMDAR, this approach may not be successful.
2. The autoantibody binding-induced conformational changes within the NMDAR. Small molecule allosteric modulators have recently been developed (5, 37, 38). Potentially, these compounds may be used to block the conformational changes induced by GluN1-aAb^pH−rhod^ binding and thus inhibit internalization.
3. Inhibition of the GluN1-aAb^pH−rhod^-induced internalization. To achieve this, general cellular trafficking pathways have to be blocked. It is questionable if such an approach can be tolerated by patients and would not cause severe side effects.

Taking these considerations into account, development and use of small molecule allosteric modulators may represent a group of drug candidates for anti-NMDAR encephalitis and other forms of autoimmune encephalitis. Evidenced by the relatively robust novel assay, they may be used to screen for compounds that block autoantibody-induced NMDAR cross-linking and internalization. This should always be accompanied by NMDAR stabilization within the synapse to avoid accumulation of NMDAR at extra-synaptic sides of the cell membrane potentially promoting excitotoxic cell death in NMDAR encephalitis.

Therefore, screening results obtained with our assay in HEK 293T overexpressing NMDAR should always be validated using super-resolution microscopy in cultured living neurons and brain slices exhibiting physiological expression levels and subcellular localization of NMDARs.

## Conclusion

This novel assay allows to unravel molecular mechanisms of autoantibody-induced receptor internalization and to study novel small-scale specific molecular-based therapies for autoimmune encephalitis syndromes.

## Data Availability

The dataset obtained and analyzed in the current study is available from the corresponding author on a reasonable request.

## Author Contributions

CS and AD: collected patient samples under the supervision of HW, SM, and NM; SBa and NG: performed the synthesis and pH-rhodamine labeling of GluN1-aAb^pH−rhod^; EA, SBe, SP, and JS: performed the transfection, immunocytochemistry, and confocal microscopy with the HEK 293T cells; EA, CB, and TS: together with TB, NS-S, GS, and NM performed incubation of transfected HEK 293T cells with GluN1-aAb^pH−rhod^ and data analysis; H-PH, BW, SM, GS, and NM: designed and supervised the project; EA, SM, GS, and NM: wrote the first draft of the manuscript. All authors contributed to and approved the final version of the manuscript.

### Conflict of Interest Statement

The authors declare that the research was conducted in the absence of any commercial or financial relationships that could be construed as a potential conflict of interest.

## Abbreviations

AMPA: α-Amino-3-hydroxy-5-methyl-4-isoxazolepropionic acid
AMPAR: α-Amino-3-hydroxy-5-methyl-4-isoxazolepropionic acid receptor
CNS: central nervous system
DMEM: Dulbecco's modified Eagle's medium
DN: dominant-negative
ECFP: enhanced cyan fluorescent protein
EE: early endosomes
EGFP: enhanced green fluorescent protein
ER: endoplasmic reticulum
FBS: fetal bovine serum
GluN1-aAb^pH−rhod^: pH-rhodamine-labeled human recombinant monoclonal GluN1 IgG1 autoantibody
GA: Golgi apparatus
GC: Golgi complex
GluR: glutamate receptors
Ig: immunoglobulin
iGluR: ionotropic glutamate receptor
KAR: kainate receptor
NEAA: non-essential amino acids
NMDA: N-Methyl-D-aspartate
NMDAR: N-Methyl-D-aspartate receptor
NT: neurotransmitter
mEPSCs: miniature excitatory post-synaptic currents
mGluR: metabotropic G-protein coupled receptor
PSD-95: postsynaptic density protein 95
RAB: Ras-related in brain
RE: recycling endosomes
RV: recycling vesicle
WT: wild-type.
